# Crowding-induced morphological changes in synthetic lipid vesicles determined using smFRET

**DOI:** 10.3389/fbioe.2022.958026

**Published:** 2022-10-28

**Authors:** Steven D. Quinn, Lara Dresser, Sarah Graham, Donato Conteduca, Jack Shepherd, Mark C. Leake

**Affiliations:** ^1^ School of Physics, Engineering and Technology, University of York, York, United Kingdom; ^2^ York Biomedical Research Institute, University of York, York, United Kingdom; ^3^ Department of Biology, University of York, York, United Kingdom

**Keywords:** single-molecule, TIRF, FRET, membrane mechanics, lipid vesicle, molecular crowding

## Abstract

Lipid vesicles are valuable mesoscale molecular confinement vessels for studying membrane mechanics and lipid–protein interactions, and they have found utility among bio-inspired technologies, including drug delivery vehicles. While vesicle morphology can be modified by changing the lipid composition and introducing fusion or pore-forming proteins and detergents, the influence of extramembrane crowding on vesicle morphology has remained under-explored owing to a lack of experimental tools capable of capturing morphological changes on the nanoscale. Here, we use biocompatible polymers to simulate molecular crowding *in vitro*, and through combinations of FRET spectroscopy, lifetime analysis, dynamic light scattering, and single-vesicle imaging, we characterize how crowding regulates vesicle morphology. We show that both freely diffusing and surface-tethered vesicles fluorescently tagged with the DiI and DiD FRET pair undergo compaction in response to modest concentrations of sorbitol, polyethylene glycol, and Ficoll. A striking observation is that sorbitol results in irreversible compaction, whereas the influence of high molecular weight PEG-based crowders was found to be reversible. Regulation of molecular crowding allows for precise control of the vesicle architecture *in vitro*, with vast implications for drug delivery and vesicle trafficking systems. Furthermore, our observations of vesicle compaction may also serve to act as a mechanosensitive readout of extramembrane crowding.

## Introduction

Native biological membranes are highly complex and heterogeneous in both size and composition, motivating the development of controllable model-membrane systems whose physical and chemical parameters can be easily tuned (Chan and Boxer, 2007). A particularly valuable class of such systems are spherical synthetic vesicles, which comprise a phospholipid bilayer surface whose radius of curvature can be carefully controlled. Small unilamellar vesicles (SUVs), for example, range from *ca.* 10–100 nm in size, whereas large unilamellar vesicles (LUVs) and giant unilamellar vesicles (GUVs) range from *ca.* 100–1,000 nm and >1 μm in diameter, respectively (Chan and Boxer, 2007; Zong et al., 2022). Importantly, their phospholipid compositions can be tailored to enable variations in charge, localized membrane roughness, phase separation behavior across the bilayer, and membrane fluidity (Chan and Boxer, 2007).

Synthetic vesicles have enabled new insights into membrane mechanics (Torchilin, 2005), interactions between lipids and proteins (Stengel et al., 2008; Okumus et al., 2009), and fusion dynamics (Mora et al., 2020) and inspired the development of drug delivery vehicles due to their low toxicity, high loading capacity, and controllable release kinetics (Torchilin, 2005; Zong et al., 2022). LUVs have found particular utility as nanoscale containers for constraining biomolecules for single-molecule analysis, where inducing porosity into their membrane facilitates buffer exchange without removing the biomolecule under interrogation (Okumus et al., 2009). Additionally, vesicles can be biochemically programmed to interact, enabling lipid mixing and content exchange (Stengel et al., 2008; Diao et al., 2012; Mora et al., 2020), and the use of perturbative detergents, which alters their morphology (Dalgarno et al., 2019; Juan-Colas et al., 2020) is important in the context of lysis and for triggering the release of encapsulated molecules (Anandan and Vrielink, 2016). It is also now clear that the structure and dynamics of synthetic vesicles are influenced by factors such as molecular crowding, but new experimental approaches are required to explore these interactions further.

The living cell’s interior is a densely crowded environment, with up to 40% of the cytoplasm occupied by solubilized macromolecules (Model et al., 2021). In this tightly filled space, excluded volumes give rise to steric repulsions, depletion attractions, reduced translational degrees of freedom, biomolecular shape changes, and diffusional effects, all of which contribute to the cell’s overall function (Zimmerman and Minton, 1993). In this context, the effect of macromolecular crowding on protein (Neuweiler et al., 2007; Soranno et al., 2014) and nucleic acid (Dupuis et al., 2014; Baltierra-Jasso et al., 2015; Holmstrom et al., 2015) structures has been heavily studied *in vitro*. For example, molecular crowding influences protein stability (Minton, 2005; Sarkar et al., 2013), interactions, kinetics, diffusion (Dix and Verkman, 2008), and liquid–liquid phase separation events (Keating, 2012). These results not only lend support to the idea that molecular crowding is a regulator of biomolecular activity but also that living cells may actively regulate crowding to enhance or adjust key processes (van den Berg et al., 2017). Several different crowding mechanisms have been proposed depending on the biomolecule and co-solute, but recent theoretical studies and energy transfer experiments point toward an intriguing size dependence, where smaller molecular weight crowders are more effective than larger polymers (Culik et al., 2014; Holmstrom et al., 2015; Sung et al., 2021a). Despite such developments, however, the question of how macromolecular crowding influences the global structure of single lipid vesicles remains largely under-explored.

Initially, experiments assessing the impact of molecular crowding on the membrane involved the use of vesicles *in vitro* under relatively dilute concentrations of ions or other chemical factors. For example, crowding was observed to give rise to concentration- and polymer-dependent osmotic pressures and non-specific depletions resulting from effects assigned to excluded volumes (Money, 1989; Parsegian et al., 2000). When high molecular weight crowding agents were introduced, the preferential exclusion of macromolecules from the membrane bilayer introduced an osmotic imbalance, which in turn altered the global membrane conformation and promoted membrane fusion (Lentz, 1994; Kuhl et al., 1996). Recent studies have shown that encapsulation of crowding agents within single vesicles leads to depletion forces that result in variations in membrane topology and changes to the surface area (Chen et al., 2004; Terasawa et al., 2012). When highly hygroscopic crowders such as polyethylene glycol (PEG) were encapsulated, the membrane dehydrated, effectively leading to vesicle compression (Lehtonen and Kinnunen, 1995). Optical microscopy experiments on 20–60 μm-sized GUVs have also demonstrated that the encapsulation of high molecular weight PEGs induces membrane stress, oscillations in vesicle size, changes to the membrane tension, permeabilization, and variations in the spatial orientation of membrane-bound molecules (Su et al., 2018). Furthermore, recent investigations on GUV crowding by nucleic acids point toward the formation of local elastic deformations and transient instabilities in the membrane (Cherstvy and Petrov, 2013). These results support a model in which molecular crowding influences the architecture, dynamics, and integrity of biological membranes. However, optical microscopy experiments on GUVs, such as those reported, only reveal macroscopic changes taking place within a 2-dimensional image plane, without quantitatively reporting on molecular level changes across the entire volume. Moreover, the influence of low molecular weight crowders is currently unclear. These challenges, combined with the need to assess the structural integrity of vesicles at the opposite end of the membrane-curvature space, motivated us to extend our single-molecule Förster resonance energy transfer (smFRET) toolbox to quantitatively assess the conformational changes taking place within sub-micron-sized LUVs in response to a range of molecular crowders *in vitro*.

Here, we employed a single-vesicle assay based on measuring the extent of smFRET between lipophilic fluorophores integrated into the membrane of LUVs to evaluate their conformation in response to crowding agents in the extravesical space. We first integrated the probes into the LUV bilayer and observed conformational changes in response to crowding *via* ensemble fluorescence spectroscopy and time-correlated single-photon counting. Electron microscopy and dynamic light scattering approaches were then used to quantify structural variations before wide-field total internal reflection fluorescence microscopy was used to capture the FRET response from single vesicles. By monitoring changes to the FRET efficiency within freely diffusing and surface-immobilized vesicles, we correlate crowding-induced changes in fluorescence signals to morphological changes within single vesicles. In particular, we found that both sorbitol, a model sugar-based cosolvent for low molecular weight crowding (Yadav, 2013), and high molecular weight crowders such as PEG400, Ficoll400, and PEG8000 could enhance the observable FRET efficiency. Compaction induced by the high molecular weight crowders was found to be reversible, whereas sorbitol-induced vesicle compaction was permanent. We expect the presented tools will be widely applicable beyond the interactions studied here, and we discuss the implications of our findings for enabling control over vesicle morphology, drug delivery, vesicle trafficking, and the regulation of vesicle curvature.

## Materials and methods

### Lipid vesicle preparation

1-Palmitoyl-2-oleoyl-glycero-3-phosphocholine (POPC) and 1-palmitoyl-2-oleoyl-sn-glycero-3-phospho-L-serine (POPS) lipids in chloroform were purchased from Avanti Polar Lipids and used without any additional purification. 1,1′-Dioctadecyl-3,3,3′,3′ tetramethylindocarbocyanine perchlorate (DiI) and 1,1-dioctadecyl-3,3,3,3-tetramethylindodicarbocyanine (DiD) were obtained from Thermo Fisher Scientific. Synthetic vesicles were prepared *via* the extrusion method as previously described (Dalgarno et al., 2019; Juan-Colas et al., 2020). Briefly, mixtures of lipids and membrane stains were mixed in chloroform at final lipid concentrations of 10 mg lipid/ml. The solvent was then evaporated by nitrogen flow to create a dry lipid film, subsequently hydrated in 50 mM Tris buffer (pH 8.0), and mixed by a vortex. The resuspended solution was then extruded through a polycarbonate membrane filter to produce vesicles of appropriate diameter.

### Ensemble fluorescence spectroscopy

Fluorescence emission spectra obtained from DiI- and DiD-loaded vesicles in 50 mM Tris buffer (pH 8) were recorded using a HORIBA Fluoromax-4 spectrophotometer with λ_ex_ = 532 nm. All experiments were performed using a final lipid concentration of 25 μM. Apparent FRET efficiencies, *E*_*FRET*_, were estimated *via E*_*FRET*_ *= (I*_*A*_ */[I*_*A*_ *+ I*_*D*_ *])*, where *I*_*A*_ and *I*_*D*_ are the integrated and background-corrected fluorescence emission intensities of the donor, DiI, and the acceptor, DiD, respectively. The data points plotted represent the mean and standard deviation obtained from three separate experimental runs.

### Time-correlated single-photon counting

Time-resolved fluorescence decays were collected using a FluoTime300 time-correlated single-photon counting spectrophotometer equipped with a hybrid PMT detector (Picoquant, Germany). Decays were measured under magic angle conditions using a pulsed excitation wavelength of 532 nm at 80 MHz and emission wavelength of 565 nm for DiI–DiD loaded vesicles. Excitation of 485 nm (20 MHz) and emission of 600 nm were used for vesicles incorporating the tension reporter FliptR (Colom et al., 2018). All experiments were performed using a final lipid concentration of 25 μM in 50 mM Tris buffer (pH 8). Decays were acquired until 10^4^ counts at the decay maximum and were observed and fitted by iterative re-convolution of the instrument response function and the observed fluorescence decay using a multi-exponential decay function of the form $I_{t}=\overset{n}{\underset{i=1}{\sum }}a_{i}e^{-\frac{t}{\tau_{i}}}$ , where *I*_*t*_ is the intensity at time, *t*, and *t*_*i*_ and *a*_*i*_ represent the fluorescence lifetime and fractional amplitude of the *i*th decay component, respectively

### Dynamic light scattering

The hydrodynamic diameter, *d*_*H*_, of freely diffusing vesicles was estimated using a Zetasizer μV system equipped with a λ_o_ = 633 nm wavelength line. Briefly, the Brownian motion of LUVs in the solution gives rise to fluctuations in the intensity of backscattered light at *θ* = 178^o^. This was used to produce a correlation function, G(τ) , *via* $G^{2}\left(\tau \right)=<I\left(t\right)I\left(t+\tau \right)>/<I\left(t\right)$ (Zong et al., 2022), where *τ* is the lag time. Correlation curves were fitted to a model of the form $G\left(\tau \right)=A\left[1+Be^{-2Dq^{2}\tau }\right]$ , where *D* is the vesicle diffusion coefficient, *A* and *B* are positive constants, and $q=\frac{4\pi n}{\lambda_{o}}\sin\left(\frac{\theta }{2}\right)$ , where *n* is the refractive index of the solution (*n* = 1.33). Hydrodynamic diameters were then calculated according to the Stokes–Einstein relationship (Stetefeld et al., 2016) and reported as the mean and standard deviation from three separate experimental runs.

### Scanning electron microscopy

SEM micrographs of vesicles non-specifically bound to a silicon substrate were acquired using a JEOL JSM-7800F system operating at 5 kV (Stengel et al., 2008; Conteduca et al., 2021). Vesicles were prepared in 50 mM Tris buffer (pH 8) containing molecular crowders at the concentration specified in the main text, diluted ∼10x in deionized water, and vortexed. The vesicles were then applied to the silicon substrate, and the solution was evaporated. The substrate was then sputtered with a 5-nm-thick Pt/Pd layer to avoid charging effects and possible damage to the vesicles during the micrograph acquisition. Vesicle diameters were then determined using ImageJ, and histograms were produced using 25-nm bin widths.

### Cryo-transmission electron microscopy

Cryo-TEM was used for direct visualization of vesicle bilayers. To prepare samples for cryo-TEM analysis, Quantifoil copper R 1.2/1.3 200 mesh grids (Electron Microscopy Sciences) were prepared by glow discharging at 20 mA and 0.26 mbar for 1 min in a Pelco easiGlow system. Small volumes (2 μl) of the vesicle sample were applied to the carbon side of the EM grid in 90% humidity. Next, the liquid was blotted off for 0.5 s and the grids were plunge-frozen into precooled liquid ethane using a Vitrobot system (Thermo Scientific). This process enabled single vesicles to be embedded within a thin layer of amorphous ice, preserving them in their native state. The samples were then evaluated using a Thermo Scientific Glacios Cryo-TEM electron microscope. TEM images were acquired using an accelerating voltage of 200 kV and x120,000 magnification. The vesicle sizes were then measured using ImageJ.

### Total internal reflection fluorescence microscopy

Microfluidic flow cells were constructed as described previously (Dresser et al., 2021) and sequentially incubated with 0.1 mg/ml BSA-Biotin, 1 mg/ml BSA, and 0.2 mg/ml NeutrAvidin. After each incubation step, the flow cells were rinsed with buffer (50 mM Tris, pH 8) to remove unbound material. Biotinylated vesicles containing 0.1 mol% DiI and 0.1 mol% DiD were then added to the surface using a final concentration of 70 μg/ml lipids in imaging buffer (50 mM Tris, 6% (W/V) D-(+)-glucose containing 1 mM Trolox, 6.25 μM glucose oxidase, and 0.2 μM catalase) and incubated for 15 min at room temperature to achieve a surface coverage of ∼150–200 vesicles per 50 × 50 μm field of view. After incubation, the flow cells were rinsed with imaging buffer. TIRF microscopy was then performed on a custom-modified inverted microscope (Nikon Eclipse Ti) containing a CFI Apo TIRF 100 x NA 1.49 oil-immersion objective lens (Nikon). TIRF illumination was provided by a TEM_00_ 532-nm wavelength line (Obis, Coherent) at < 8.2 mW cm^−2^. The emission line was separated from the excitation line *via* a dichroic and emission filter mounted beneath the lens turret (Chroma 59907-ET-532/640). DiI and DiD emission line was then spatially separated using a DualView image splitter (Photometrics) containing a dichroic filter (T640LPXR, Chroma) and band-pass filters (ET585/65M and ET700/75M, Chroma) and imaged in parallel on a back-illuminated Prime 95B CMOS camera cooled to −30°C (Photometrics). After each addition of crowder in the imaging buffer, 500 frame movies were acquired with 50 ms exposure time. Recorded images were then analyzed in MATLAB (R2019a) using iSMS single-molecule FRET microscopy software (Preus et al., 2015). Briefly, the donor and acceptor emission channels were aligned, and background-corrected DiI- and DiD-integrated emission trajectories were obtained within the excitation field. Apparent FRET efficiencies across the trajectories were then calculated using $I_{A}/\left(I_{A}+I_{D}\right)$ as previously described and related to the mean distance between probes, *R*, *via* $E=\frac{R_{0}^{6}}{R_{0}^{6}+R^{6}}$ , where *R*_*o*_ is the Förster radius (5.3 nm) (Dalgarno et al., 2019). *E*_*FRET*_ histograms from *N* > 2000 vesicles were then produced using bin widths of 0.01.

## Results and discussion

### Sorbitol induces conformational changes in freely diffusing lipid vesicles

We first used smFRET between lipophilic membrane stains to explore the structure of LUVs composed of POPC lipids in the presence of synthetic molecular crowding agents. Previous studies evaluating membrane deformation have largely relied on encapsulating molecular crowders or introducing pegylated lipids into GUV bilayers, with single-color fluorescence imaging used to evaluate macroscopic changes (Garenne et al., 2020). However, such approaches do not provide detailed information at the molecular level nor do they report on vesicles smaller than the optical diffraction limit (Dalgarno et al., 2019). We, therefore, applied smFRET to quantify the nanoscale structural variations in LUVs of ∼200 nm in size in response to dilute crowding conditions.

We first optimized the number of donors (DiI) and acceptors (DiD) per LUV (1:1 ratio, 0.1 mol% of each dye), such that the average FRET efficiency (*E*_*FRET*_) per vesicle was initially ∼0.5. This corresponds to an average DiI–DiD separation close to their Förster radius and allows for nanoscale changes because of vesicle compaction or swelling to be quantified by an observable increase or decrease in E_FRET_. To mimic low molecular weight crowding, we used the polyol osmolyte sorbitol, an established low molecular weight crowding agent, which has previously been applied to regulate protein clustering (Sukenik et al., 2011; Khodadadi et al., 2014; Sukenik et al., 2015), induce nuclear organization, and compact chromatin (Richter et al., 2007).

**FIGURE 1:**
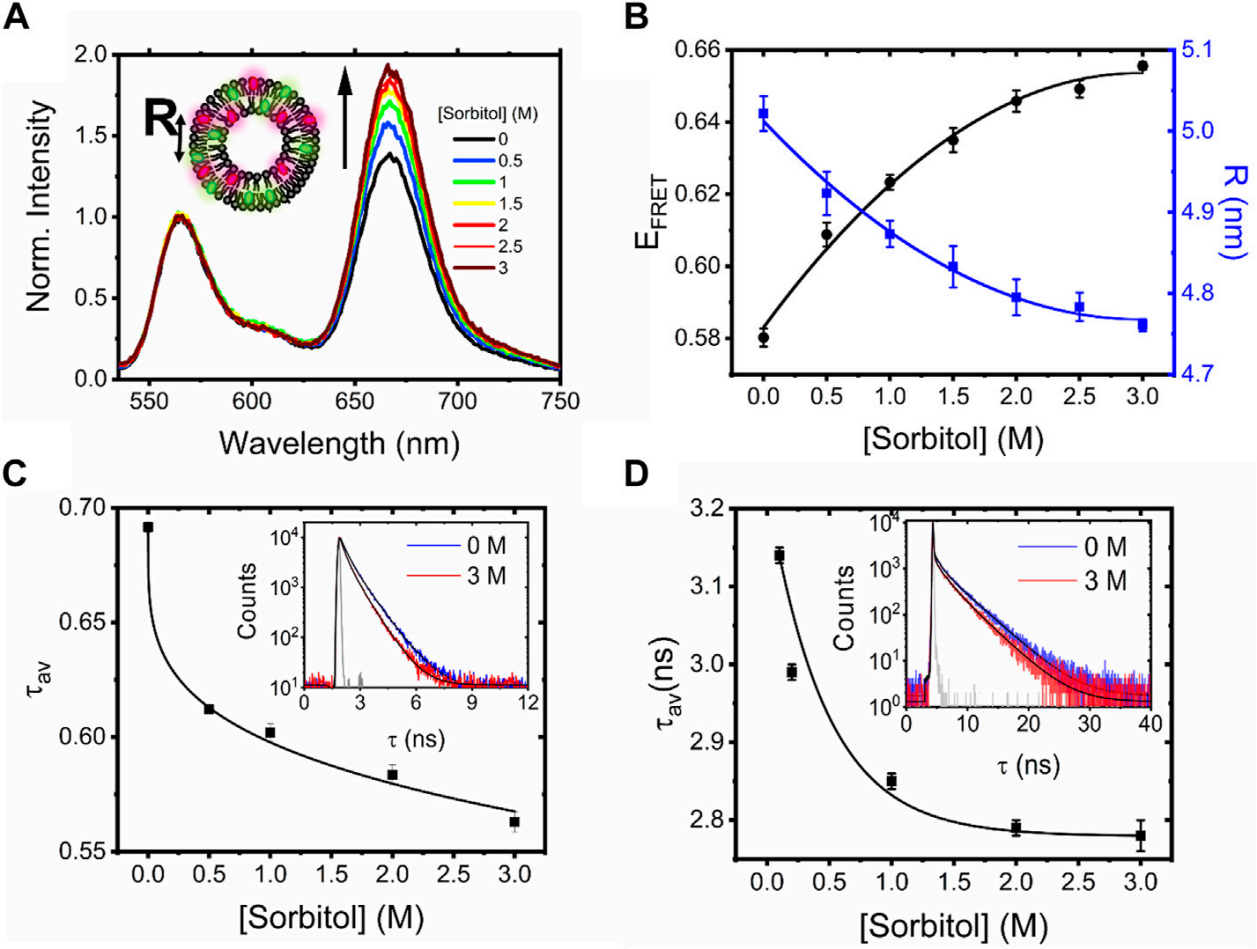
Sorbitol induces conformational changes in freely diffusing vesicles. **(A)** Normalized fluorescence emission spectra of DiI–DiD LUVs in the absence and presence of sorbitol (λ_ex_ = 532 nm). Inset: schematic illustration of the vesicles, where *R* corresponds to the mean dye–dye separation distance. **(B)** Corresponding variations in *E*_*FRET*_ and *R*. Heuristic fits shown are quadratic (solid black lines), determined with Python 3 using NumPy’s polyfit routine. **(C)** Amplitude weighted average lifetime of DiI and **(D)** FliptR as a function of sorbitol. Insets correspond to the time-resolved fluorescence decays in the absence (blue) and presence of 3M sorbitol (red). Solid black lines represent biexponential fits to the raw data, and the solid gray lines represent the instrument response functions.

Dynamic light scattering first confirmed the formation of labeled vesicles with a log-normal hydrodynamic diameter centered on 227 nm (Supplementary Figure S1). When vesicles were prepared without labels, a similar distribution was observed, providing confidence that the labeling process does not perturb morphology (Supplementary Figure S1). We then recorded the ensemble fluorescence spectra obtained from the labeled vesicles in solution. As shown in Figure 1A **,** DiI- and DiD-loaded LUVs displayed a progressive increase in sensitized acceptor emission and E_FRET_ as the sorbitol concentration progressively increased, translating to a reduction in the mean DiI–DiD separation distance from 5.02 ± 0.02 nm in the absence of crowding to 4.76 ± 0.01 nm in the presence of 3 M sorbitol (Figure 1B). To further confirm the presence of an energy transfer mechanism, we measured the amplitude-weighted fluorescence lifetime, τ_av_, of DiI in the presence of DiD *via* time-correlated single-photon counting**.** Here*,* $\tau_{av}$ decreased with increasing sorbitol concentration, consistent with a progressive quenching of DiI and a corresponding increase in E_FRET_ (Figure 1C). The decays fitted well to a bi-exponential decay after reconvolution with the instrumental response function, which we assigned to energy transfer between dyes on the inner and outer leaflets. Indeed, a bootstrap analysis revealed that both lifetime components, termed $\tau_{1}$ and $\tau_{2}$ , progressively decreased with increasing sorbitol concentration (Supplementary Figure S2), suggestive of global conformational changes on both sides of the membrane. Taking these measurements together, the data pointed toward fluorophore packing in the ensemble and were suggestive of vesicle compaction.

To test whether sorbitol induced variations in membrane tension, we next evaluated changes in the fluorescence lifetime of 1 mol% Fluorescent LIPid Tension Reporter (FliptR) incorporated within the LUV bilayer. The FliptR lifetime correlates well with membrane tension (Colom et al., 2018), and in our case, we found that decays fitted well to a biexponential model. In line with previous observations, the longer lifetime component, *τ*_*2*_, only accounted for a small fraction of the overall signal. In the absence of sorbitol, the ensemble FliptR lifetime was found to be 3.14 ± 0.01 ns, corresponding to a situation where the molecules reside in a planar conformation. However, as sorbitol was progressively titrated, we observed a reduction in the lifetime to ∼90% of its original value (Figure 1D, Supplementary Figure S3), which we hypothesize is due to a fraction of FliptR molecules twisting into a conformation that is sterically more favorable because of changes to the lipid packing density and decreased membrane tension. Similar to observations made with single vesicles in response to surfactants (Drab et al., 2021), we speculate that phospholipid bilayers mixed well with sorbitol lead to a situation where bilayer components are forced by entropy, resulting in mixed sorbitol–lipid aggregates, local membrane undulations, and reduced membrane tension.

The ensemble FRET, lifetime, and FliptR analysis broadly supports conformational rearrangements taking place in LUVs in response to sorbitol. However, to understand the parameters that affect this process, we next moved to interrogate the impact of vesicle size, composition, and phase. When vesicles of various sizes (100, 400, and 1,000 nm), as confirmed by DLS (Supplementary Figure S4), interacted with sorbitol, the FRET efficiencies in all cases increased with crowder concentration, signifying similar dye–dye distance changes (Supplementary Figure S5). Indeed, the relative magnitude of the FRET enhancement compared to that observed from 200-nm diameter vesicles was similar across all conditions tested.

To assess the impact of lipid composition and phase, we next probed the interaction between sorbitol and 100-nm diameter vesicles containing POPS lipids as a function of temperature and compared the relative change in FRET efficiency to similarly sized vesicles composed of POPC (Supplementary Figure S5). POPC has a gel-to-liquid phase transition temperature, *T*_*M*_, of −2°C and is therefore in the liquid phase at temperatures > 4°C, whereas *T*_*m*_ = 14°C for POPS, below which the vesicles are in the gel phase. We observed that the initial FRET efficiency magnitude was similar for both sets of vesicles at 4°C, 21°C, and 37°C; however, the relative change in E_FRET_ for each sorbitol condition was generally larger in the case of POPC vesicles. One possible explanation for the observed difference could rest in the hydration of the lipid carbonyls. POPS lipids are less mobile and give rise to dehydrated vesicle forms (Jurkiewicz et al., 2012), suggesting that the uptake of sorbitol into the bilayer may be key for conferring the observed changes.

### Morphological characterization of single vesicles in response to sorbitol

**FIGURE 2:**
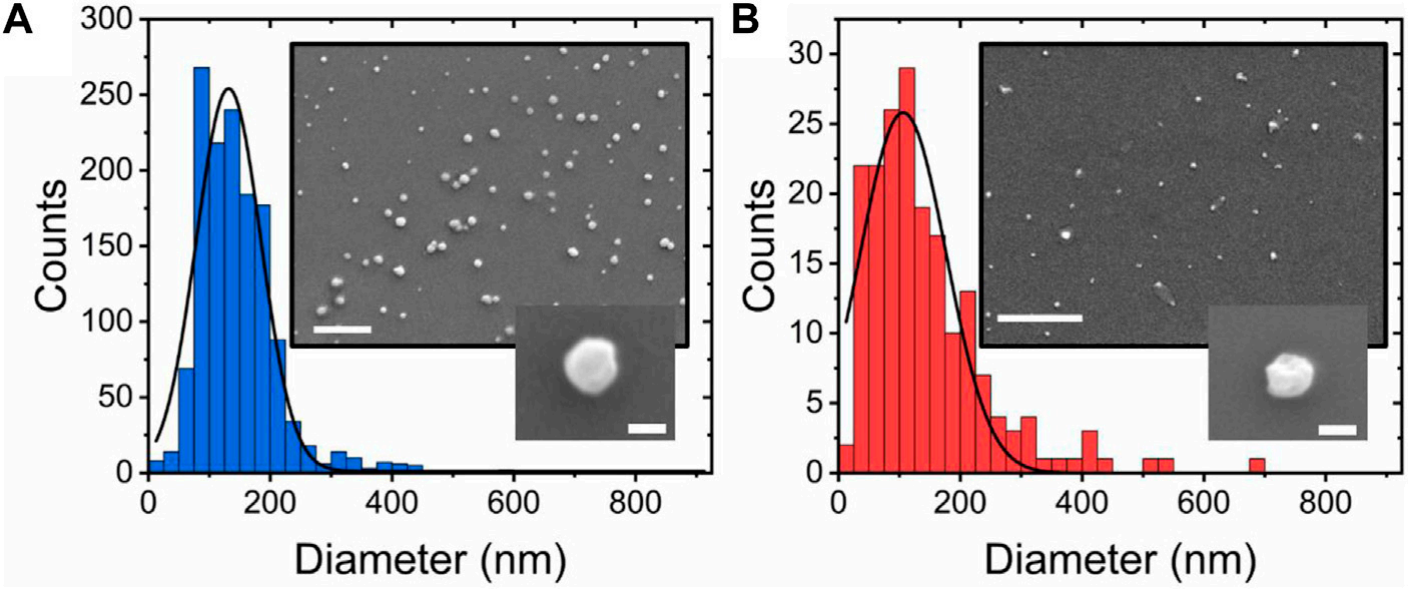
Sorbitol induces compaction and undulations in single-lipid vesicles. Quantitative comparison of diameter distributions of POPC vesicles in **(A)** the absence and **(B)** presence of 3 M sorbitol. Insets: representative SEM images of immobilized vesicles under the respective conditions. Scale bars = 1 μm and 100 nm in the larger and smaller insets, respectively.

To explore whether the observed FRET changes were coupled with changes in vesicle morphology, we next investigated the vesicle sizes using scanning electron microscopy (SEM). As shown in Figure 2A, vesicles composed of POPC lipids in the absence of sorbitol were mostly spherical (circularity = 0.87 ± 0.16), in line with previous observations (Conteduca et al., 2021), with a size distribution centered on 132 ± 3 nm (FWHM = 125 ± 8 nm). However, with the addition of sorbitol, the peak shifted to 105 ± 5 (FWHM = 155 ± 15 nm) (Figure 2B) and the circularity reduced to 0.55 ± 0.12, consistent with a model in which sorbitol leads to local undulations in membrane morphology and overall compaction. This analysis was further supported by variations in the vesicle hydrodynamic diameter (*d*_*H*_) reported by DLS, where the size distributions in a solution progressively decreased upon sorbitol addition (Supplementary Figure S6). We note that the vesicle sizes reported by DLS are generally larger than those reported by SEM, likely due to the vesicles being dehydrated and fixed under vacuum for SEM imaging. Nevertheless, both measurements pointed toward sorbitol-induced structural changes taking place within single vesicles.

**FIGURE 3:**
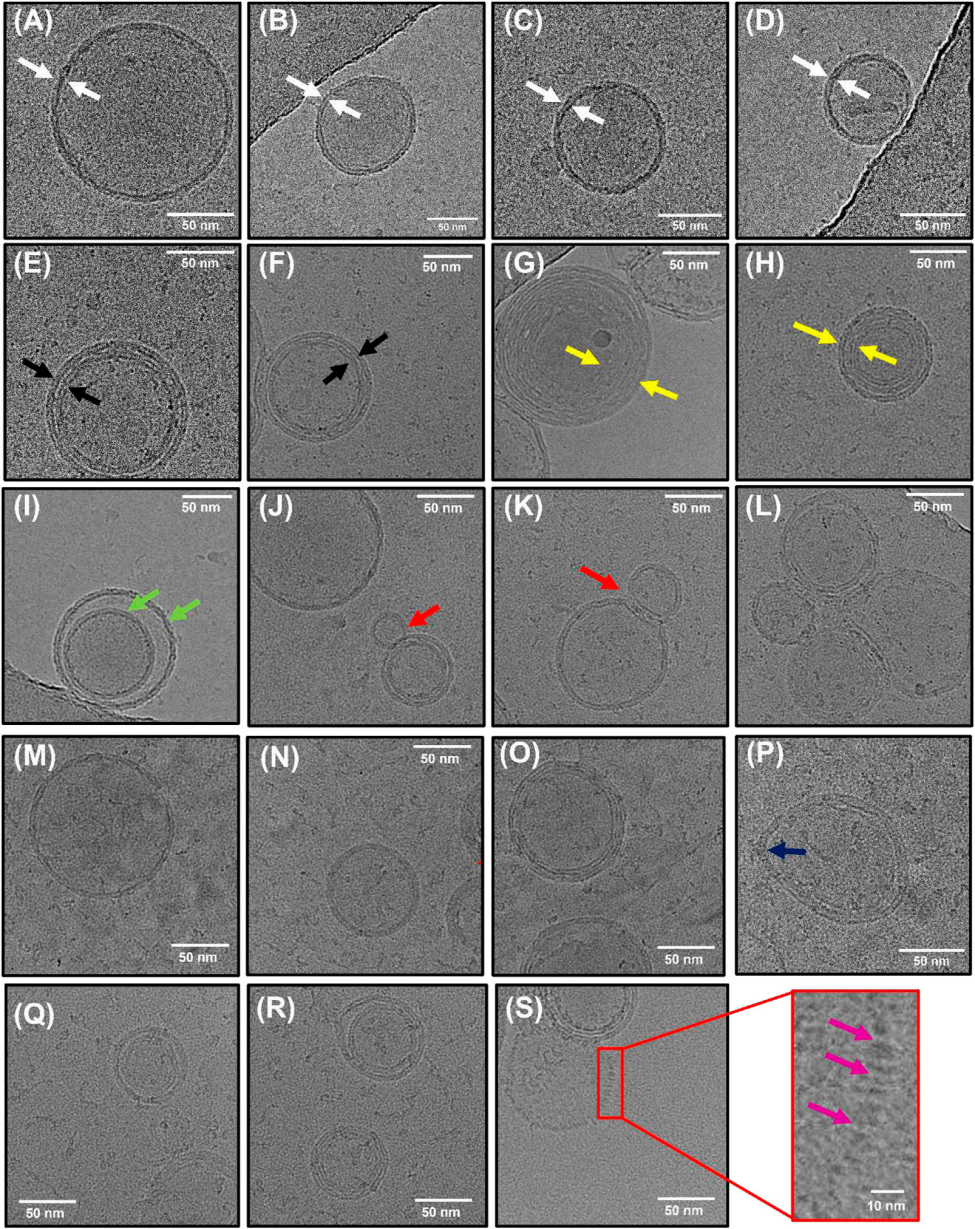
Morphological characterization of lipid vesicles by Cryo-TEM. Representative examples of **(A–D)** unilamellar (white arrows), **(E–F)** bilamellar (black arrows), and **(G–H)** multilamellar (yellow arrows) vesicles in the absence of sorbitol. Also shown are examples of **(I)** encapsulated vesicles (green arrows), **(J–K)** bowling pin-shaped vesicles, and **(L)** large conglomerates. **(M–O)** Representative examples of vesicles in the presence of 0.5 M sorbitol and **(P)** example of vesicle displaying irregular bulging. **(Q–R)** Representative examples of vesicles in the presence of 1 M sorbitol with **(S)** examples of regions of membrane damage (purple arrows).

To further characterize the morphology of the vesicles, we also performed cryo-transmission electron microscopy (cryo-TEM). This enabled us to visualize the internal structures of hydrated vesicles in a frozen state, bypassing the requirement for dehydration. In the absence of crowding agents, the majority of vesicles were classified as unilamellar, exhibiting only a single bilayer (Figures 3A–D), with lower fractions (<20%) containing double (Figures 3E,F) or multiple layers (Figures 3G,H). All of the vesicles were intact, lacked any evidence of pore formation and in line with our SEM analysis, and they were spherical in nature with diameters in the range of 60–315 nm. Among the observed variants, we also observed larger vesicles containing encapsulated smaller vesicles (Figure 3I) unusually shaped structures in the form of a bowling pin (Figures 3J,K), and large conglomerates (Figure 3L). Interestingly, the structures produced from our synthetic vesicle preparation have a striking resemblance to those observed from extracellular vesicles isolated from cerebrospinal fluid (Emelyanov et al., 2020), further validating our systems as excellent membrane mimetics. Of those vesicles classified as unilamellar, we observed a mean particle size of 157 ± 5 nm, with a membrane thickness of 6 ± 0.7 nm (*N* = 84) (Supplementary Figure S7). In the presence of 0.5 M sorbitol, the observed species were structurally similar (Figures 3M,N,O), with some vesicles displaying evidence of irregular membrane undulations and bulging (Figure 3P). However, in line with our SEM and smFRET analysis, the vesicle size distribution shifted to smaller values and the unilamellar vesicle size reduced by ∼30%–115 ± 5 nm (*N* = 138). When 1 M sorbitol was introduced, most vesicles were intact (Figures 3Q,R); however, others displayed signs of damaged, porous membranes, as indicated by the lack of a visible bilayer and lack of electron-dense material spaced across the projection of the vesicle surface (Figure 3S), and the particle size reduced further to 82 ± 5 nm (*N* = 24). Based on this evidence, we suggest that the synthetic lipid vesicles studied in this work undergo global morphological changes upon sorbitol addition that broadly involves vesicle compaction coupled with membrane undulations, direct membrane damage, and formation of pores.

### Sorbitol-induced compaction of surface-tethered vesicles is irreversible

**FIGURE 4:**
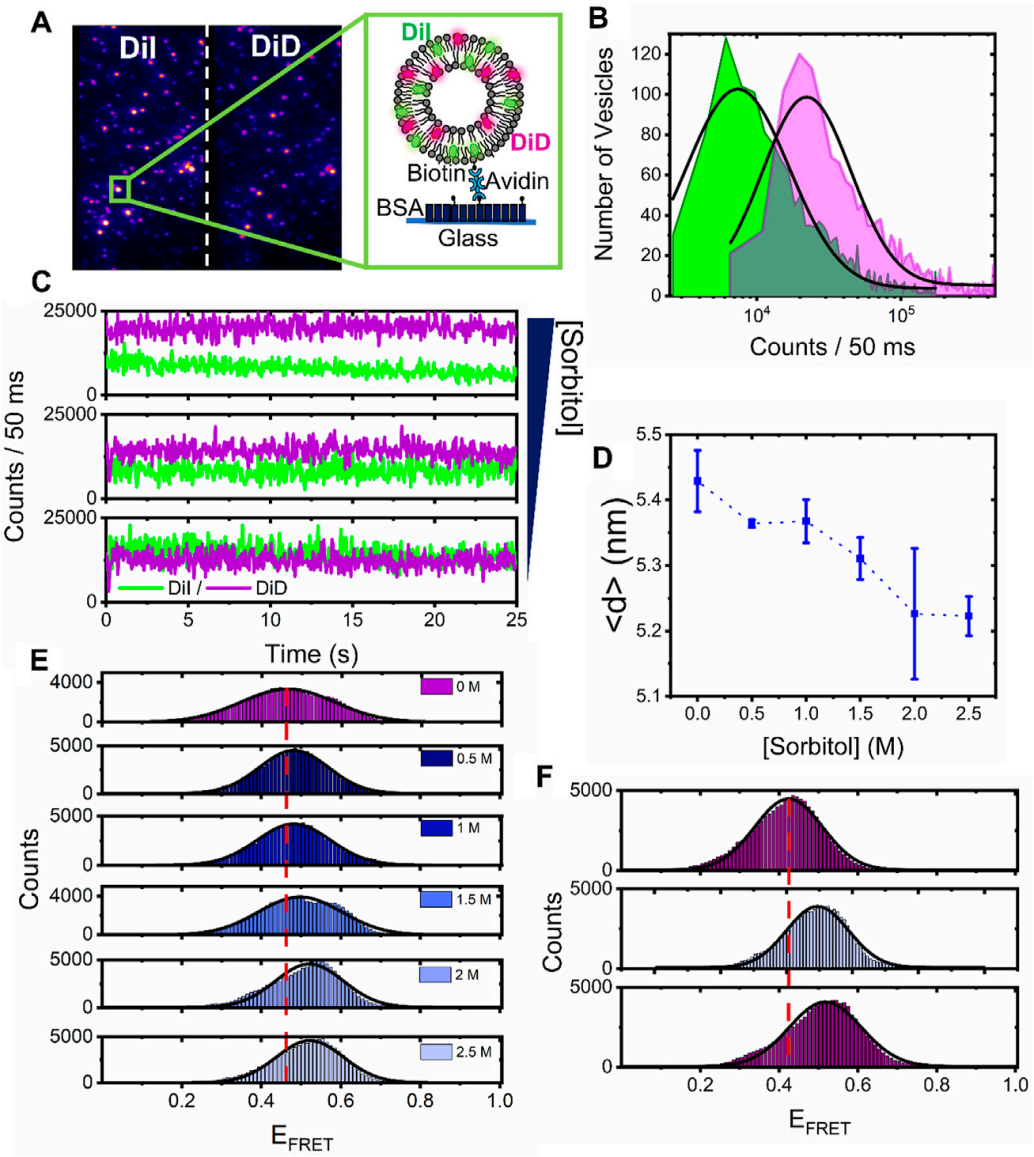
Sorbitol induces irreversible structural changes in single surface-tethered vesicles. **(A)** Representative wide-field TIRF image of surface tethered vesicles composed of DiI and DiD. Donor and acceptor emission channels are shown on the left- and right-hand side of the dashed line, respectively. Inset: surface immobilization scheme. Single vesicles containing biotinylated lipids are immobilized onto a BSA-Biotin coated glass coverslip *via* NeutrAvidin. **(B)** Fluorescence intensity population histograms of DiI (green) and DiD (magenta), obtained from surface-tethered vesicles in the absence of sorbitol. **(C)** Representative time traces of DiI (green) and DiD (magenta), obtained from single surface-tethered vesicles with 0 mM, 1 M, and 3 M sorbitol. **(D)** Representative variation in the peak probe separation distance, <*d*>, as a function of sorbitol concentration. Also shown are fitting errors associated with the application of single Gaussian distributions to histograms of the probe-separation distance. **(E)** Corresponding variations in the FRET efficiency histograms obtained for *N* > 2,000 vesicles. **(F)** FRET efficiency histograms obtained from *N* > 2,000 vesicles at 0 mM sorbitol, after a 3 M sorbitol rinse step (middle panel), and after vigorous washing of the sample with imaging buffer (lower panel). The dashed red lines in **(E)** and **(F)** correspond to the peak positions of the FRET efficiency histograms obtained in the absence of sorbitol. The solid black lines represent single Gaussian fits.

To further visualize the observed compaction events, we next evaluated the mean FRET response from single surface-tethered vesicles under crowding conditions *via* a custom-modified wide-field objective-type total internal reflection fluorescence microscope. Prior to extrusion, the lipid suspension contained 1 mol% of biotinylated lipids (Biotin-PE), allowing the formed vesicles to be tethered to a glass substrate *via* NeutrAvidin (Juan-Colas et al., 2020). Application of picomolar vesicle concentrations to a NeutrAvidin-coated surface led to the detection of 195 ± 14 FRET-active vesicles per 25 × 50 μm, with a mean nearest-neighbor vesicle separation distance of ∼1 μm (Figure 4A). In the absence of sorbitol, the vesicles displayed fluorescence across donor and acceptor emission channels, indicative of substantial FRET due to their close proximity. DiI and DiD intensity distributions obtained from *N* = 1,566 foci displayed log-normal behavior (Figure 4B), which likely represents a distribution of vesicle sizes on the surface, consistent with our EM analyses (Kunding et al., 2008; Balomenos et al., 2021). As sorbitol was progressively added, we then recorded variations in the FRET efficiency per vesicle *via* changes to the DiI and DiD emission. Specifically, we observed a progressive increase in sensitized acceptor emission as crowding was increased because of enhanced FRET between the dyes (Figure 4C). During the titration, the number of foci per field of view and the mean total fluorescence intensity per vesicle, defined as the sum of DiI and DiD emission intensities, remained largely unchanged, providing confidence that sorbitol addition left the vesicles intact on the surface. In the absence of sorbitol, the FRET efficiency distribution displayed Gaussian behavior and was centered on 0.45, corresponding to a mean DiI–DiD separation distance of 5.4 nm. With increasing levels of sorbitol, the peak of the distribution then shifted by 15% to 0.53 at 2.5 M, corresponding to a reduction in the mean DiI–DiD separation (Figure 4D). Given that the total intensity of the foci remained largely invariant upon sorbitol addition, the positive shift in the FRET efficiency population distributions observed as sorbitol was added (Figure 4E) could not, therefore, be attributed to lipid loss or photophysical artefacts, but rather to structural alterations within single vesicles, where the mean donor–acceptor separation distance, <*d*>, progressively reduced. The crowding-induced morphological changes observed here are thus consistent with compaction, and the data are broadly complementary to those of previous observations where encapsulated molecular crowders led to vesicle bulging (Su et al., 2018). In our case, the progressive addition of sorbitol led to instantaneous morphological changes on our measurement timescale. Furthermore, we observed that the compaction by sorbitol was irreversible. As shown in the top panel of Figure 4F, the vesicles were initially prepared in the absence of sorbitol and had a FRET distribution centered on 0.42. After incubation with 2.5 M sorbitol, a positive shift in the FRET distribution was observed (Figure 4F, middle panel); however, after rinsing the flow cell with buffer, the distribution post sorbitol addition remained unchanged (Figure 4F, bottom panel). This observation rules out an excluded volume effect as the underlying cause of the compaction since freely diffusing sorbitol was removed from the solution. Instead, this observation points toward a situation where after interaction of the membrane with sorbitol, the phase transition temperature is irreversibly depressed in a dose-dependent manner *via* an interaction that we speculate could involve sorbitol interactions with the functional phosphate groups on the lipids.

**FIGURE 5:**
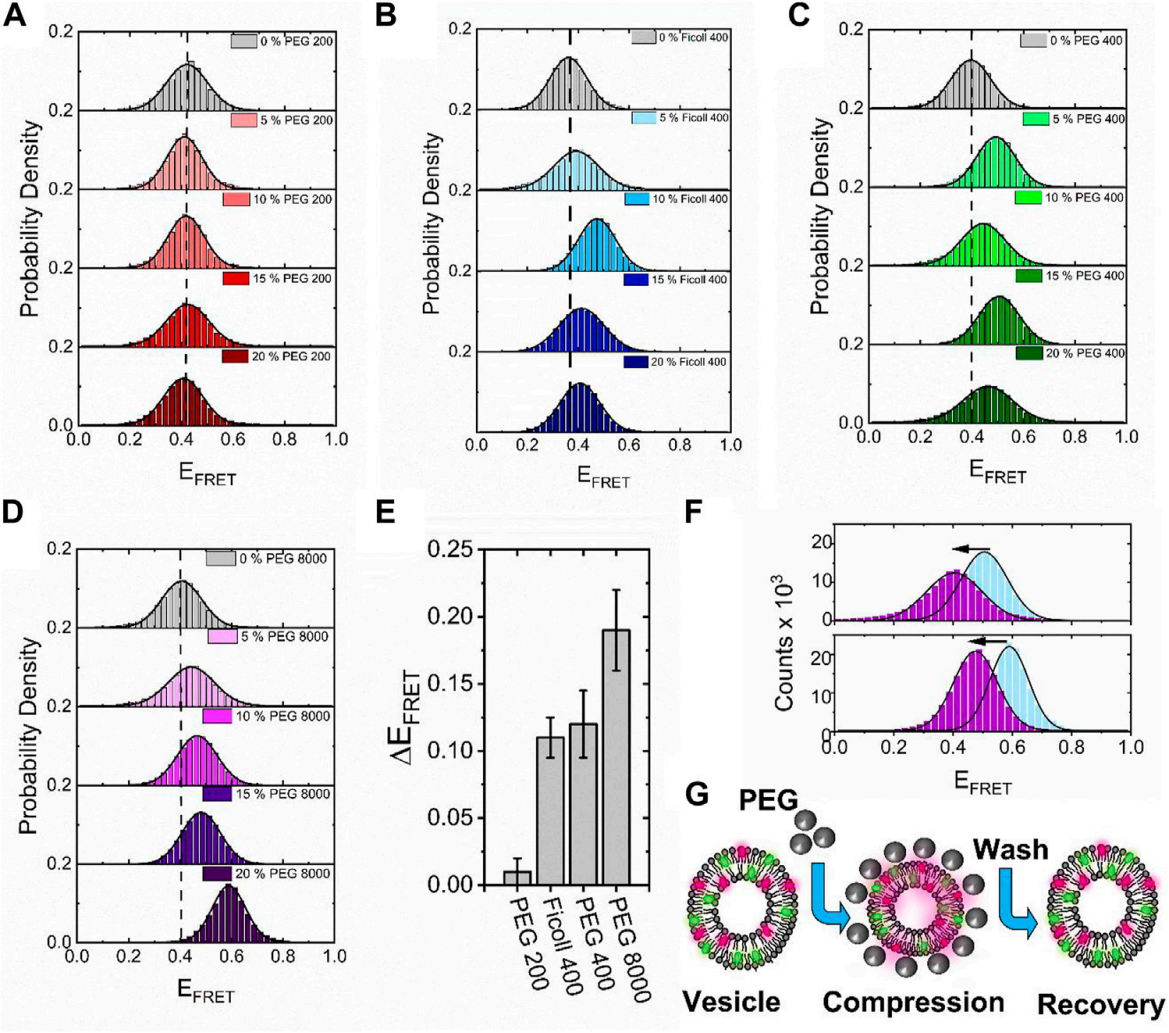
High molecular weight crowders induce reversible vesicle compaction. Representative variations in the FRET efficiency histograms obtained for *N* > 2,000 vesicles in the absence and presence of **(A)** PEG 200, **(B)** Ficol 400, **(C)** PEG 400, and **(D)** PEG 8000 at 5%, 10%, 15%, and 20% (*w/w*) in 50 mM Tris buffer (pH 8), respectively. The dashed lines correspond to the peak positions of the FRET efficiency histograms obtained in the absence of a crowder. The solid black lines represent single Gaussian fits. **(E)** Comparative bar plot summarizing the maximum variation in FRET efficiency observed under different crowding conditions. **(F)** Representative FRET efficiency histograms obtained from *N* > 2,000 vesicles in the presence of PEG400 (blue, top panel) and PEG8000 (blue, lower panel) and after vigorous washing with 50 mM Tris (pH 8) buffer (purple). **(G)** Injection of high molecular weight PEG crowders leads to reversible vesicle compaction.

Having established the DiI–DiD FRET pair as a sensitive indicator of vesicle compaction, we next explored the effect of higher molecular weight crowding agents known to induce excluded volume effects on biomolecular morphology. Here, we evaluated the fluorescence response of surface-immobilized vesicles labeled with DiI and DiD in response to variations of PEG and Ficoll. As reported previously, macromolecular crowding by PEG and Ficoll, even at modest concentrations in solution, can trigger substantial excluded volume effects, regulate mesoscale biological functions, and impact the conformations of single biomolecules (Baltierra-Jasso et al., 2015; Banerjee et al., 2016; Paudel et al., 2018; Junker et al., 2019; Kaur et al., 2019). We, therefore, hypothesized that high molecular weight crowders in the extravesicular space could influence the structural integrity of single lipid vesicles. By using the same immobilization strategy and conditions as described previously, we imaged the vesicles under low-excitation TIRF conditions with 50 ms time integration before and after the addition of molecular crowders. In the absence of crowding agents, the FRET efficiency distributions obtained from *N* > 2000 vesicles displayed Gaussian behavior centered on 0.4, corresponding to <*d*> =5.7 nm (Figures 5A–D). When 5%–20% (*w/w*) of the low molecular weight crowder PEG 200 (a 200 Da grade of PEG) was injected, the distributions remained largely invariant, indicative of little-to-no effect on vesicle morphology (Figure 5A). However, when similar experiments were performed with the highly branched Ficoll 400 and linear PEG 400, both of which represent 400 Da crowding agents, we observed positive shifts in the population histograms, with <*d*> decreasing to 5.4 nm at 10%–15% (*w/w*) crowder (Figures 5B,C). When PEG 8000 was introduced, the FRET distribution shifted further, peaking at 0.6 at 20% (*w/w*), with <*d*> =4.9 nm (Figure 5D). On the basis of spherical vesicle morphologies, the change in the inter-dye distance was observed from the vesicles in the presence of Ficoll 400, PEG 400, and PEG 8000 scales directly with the vesicle radius, and the change is thus supportive of compaction. The maximum FRET efficiency shift observed across the titration displayed a dependence on crowder size in the order PEG 8000 > PEG 400 > Ficoll 400 > PEG 200 (Figure 5E), suggesting that high molecular weight polymers are more effective at inducing this morphological change. Although the exact nature of these variations requires further investigation, our data suggest that the DiI–DiD FRET pair is sensitive to the three-dimensional structure of the vesicle and that changes to the FRET signal may arise from an excluded volume effect, with the higher molecular weight polymers leading to more pronounced changes in morphology. A particularly striking observation was that in the cases of PEG 400 and PEG 8000, the FRET efficiency distributions recovered to their original state after the vesicles were thoroughly washed with buffer solution (Figure 5F), indicating reversibility. Unlike in the case of sorbitol, where we speculate that direct sorbitol–lipid interactions lead to membrane dehydration and irreversible compaction, here, we speculate crowding arises primarily through an excluded volume effect. By removing the crowding agents from solution, we effectively remove this influence and thereby allow the vesicles to recover to their original form, as schematically shown in Figure 5G.

A direct quantitative comparison between the effects of molecular crowding, seen here on highly curved LUVs, and previous work on GUVs is not entirely straightforward. For instance, the optical imaging of GUV encapsulating molecular crowders as performed previously (Su et al., 2018) only reports on macroscopic changes from a cross-sectional slice across the vesicles. In contrast, the smFRET approach described here provides access to the mean dye separation distance across the entire three-dimensional volume of the vesicle. Nevertheless, both sets of vesicles undergo substantial morphological changes in response to molecular crowders at similar concentrations, and thus our smFRET work on sub-micron-sized vesicles is complementary to the optical imaging of GUVs, with both datasets suggesting that membrane conformation is strongly influenced by molecular crowding. While the smFRET approach provides details of vesicle perturbation on the nanoscale, a limitation is that it does not quantitatively report on pore formation or solution exchange across the bilayer. Recent studies on GUVs have provided evidence to support the transient formation of nanoscale pores induced by osmotic pressure and variations in membrane tension (Shibly et al., 2016; Billah et al., 2022; Malik et al., 2022). While we obtained limited evidence for the formation of pores induced by sorbitol (Figure 3S), whether this is also true for the sub-micron-sized vesicles in response to PEG-based crowders remains an open question. We expect the smFRET approach to play a key role in identifying the conformational changes within single vesicles in response to a wide variety of complex crowding conditions, including various solvents, solutes of varying molecular weight, composition, concentration, temperature, pH, and viscosity.

## Conclusion

In this work, we characterized the response of synthetic sub-micron-sized vesicles to environmental crowding conditions and found that both sorbitol and polymers of PEG and Ficoll influence their lipid packing density and size, even at modest concentrations of the crowder. We conclude that molecular crowding leads to global compaction of the intact vesicle structure, with our hypothesis supported by the direct observation of single vesicles by electron microscopy. Given the variability of molecular crowding *in vivo*, we subjected the vesicles to a range of molecular weight crowders, and unlike the effects of crowding on nucleic acids, which indicate smaller molecules induce more pronounced crowding effects (Sharp, 2015; Sung et al., 2021b), our observations point toward an intriguing size dependence. On one hand, we observed that crowding by the low-molecular-weight sorbitol leads to permanent vesicle compaction *via* a biophysical mechanism that likely involves the integration of the crowder into the vesicle bilayer and membrane dehydration. In contrast, crowding by longer polymers of PEG and Ficoll induced reversible vesicle compaction, likely *via* an excluded volume effect (Minton, 1981; Minton, 2001; Kilburn et al., 2010; Sung et al., 2021a), with the observed structural changes found to be more pronounced with higher molecular weight crowding agents. It should, however, be noted that these observations were made exclusively on phosphocholine-based vesicles, and future work is required to disentangle the influence of lipid composition and cholesterol content. Whether the presented FRET sensor can also be adapted to sense morphological changes to extracellular vesicles *in vivo* is yet to be explored, but we note that morphological compaction has been observed here in synthetic vesicles with similar compositions, sizes, and structures.

Understanding the structural stability of sub-micron-sized vesicles, especially those with high curvature, and how they dynamically respond to molecular crowders has a number of implications. First, important trafficking pathways between the endoplasmic reticulum rely on the formation of highly curved vesicles, and regulation of extramembrane crowding may be one mechanism through which vesicles alter morphology for vital processes, including endocytosis and vesicle budding (Shibata et al., 2009; Kozlov et al., 2014; McMahon and Boucrot, 2015). More generally, crowding-induced changes to local membrane curvature could also play a role in the shaping of organelles and the triggering of adaptive cellular responses such as gene expression (Pittman et al., 2022). Second, our measurements indicate that extramembrane crowding leads to variations in the lipid packing density, which does not always guarantee stability (van der Koog et al., 2021). In the context of vesicles as drug delivery vehicles, it is possible that molecular crowding influences the efficiency at which encapsulated drug molecules are released and, therefore, the fate of the targeted cell. Finally, the ability to precisely control vesicle morphology is highly desirable for a number of biotechnological applications (Guerzoni et al., 2022). For instance, we foresee that the ability to alter vesicle curvature *in vitro* could open a platform for investigating crowding effects on membrane protein signaling and for evaluating encapsulation efficiencies in more physiologically relevant conditions.

## Data Availability

The datasets presented in this study can be found in online repositories. The names of the repository/repositories and the accession number(s) can be found below: Data associated with this study are freely available from DOI:10.5281/zenodo.7081992.
